# The Evolution of Paediatrics
*An address to the Bath Clinical Society on 13th November, 1959.


**Published:** 1960-10

**Authors:** John Apley

**Affiliations:** Consultant Paediatrician, United Bristol Hospitals and Bath Clinical Area; Shaw Lecturer in Diseases of Children, University of Bristol


### THE EVOLUTION OF PAEDIATRICS*

BY

JOHN APLEY, M.D., F.R.C.P.,

*Consultant Paediatrician, United Bristol Hospitals and Bath Clinical Area; Shaw Lecturer in Diseases of Children, University of Bristol*

The City of Bath has known many famous men. Today I pay tribute to the Reverend Thomas Robert Malthus. He first came to Bath as a schoolboy, though he learnt little but Latin and good behaviour from his teacher, the Rector of Claverton. When he returned many years later (in 1834), to visit his wife's family, Malthus had achieved lasting fame. He died suddenly of heart disease, and was buried in Bath Abbey. An inscription to him can be seen just inside the door on the left of that gracious honey-coloured building. It describes his integrity, sweetness of temper, benevolence and piety; yet paradoxically, because of his views on the limitation of populations, he was "the best abused man of his age".

Malthus played a vital part in Darwin's theory of evolution. He described the trend of populations to increase faster than their food supply. When Darwin read Malthus's work it gave him the essential clue to natural selection: if species produce too many offspring to survive, the weakest will die and the strongest live. And, to come to my subject, because this year is the centenary of Darwin's *The Origin of Species* I have been stimulated to consider paediatrics from the evolutionary aspect.

Before I go on to discuss paediatrics in terms of evolution let me first briefly continue the Malthusian theme into our own day. It is estimated that in Biblical times the population of the whole world was about the same as is that of the United Kingdom now. Last year the world population *increased* by approximately the same number. The rate of increase is accelerating, but in the world as a whole the production of food is not keeping pace.

Can this Malthusian dilemma be solved? It might eventually be solved by "fertility control", as it has been called. There are already oral methods of contraception for women; and the sterilization of men by vasectomy is practised in at least one over-populated country in the East. From the opposite approach, the production of food may increase startlingly, by photosynthesis, for example, or by the harvesting of the oceans. Will these methods solve the problem, or merely postpone its solution?

An eminent scientist recently suggested that the population problem is likely to be solved catastrophically. He and others—may I dub them Malthusiasts?—forecast that a population "crash" is imminent. One of three outcomes is anticipated. A trigger-happy politician with a large store of nuclear weapons may abolish at the same time our worries about population and most of the population itself. Or a virulent infective agent may appear, like myxomatosis in rabbits. Or we may be overwhelmed by a "stress syndrome", like that which affects voles and jack-rabbits in periods of over-population.

To us as doctors possibly the most immediately relevant aspect of Darwin's theory is the law of adaptation. Species or sub-species adapt themselves to their environment: they adapt or perish. I am going to apply this tentatively to paediatrics, which may be considered one of the latest sub-species of medicine; for a sub-species is a section of a species that has become sufficiently differentiated to deserve a name of its own, but can still inter-breed with the rest of the species. In retrospect, the course of

* An address to the Bath Clinical Society on 13th November, 1959.

change in successful evolution appears to be not haphazard but directional; there is progress, usually from simple to complex and from lesser to greater efficiency. This certainly applies to paediatrics. Again, the evolution of vertebrates goes with a continuous exploration of new environments: paediatricians with a backbone are continually exploring new environments.

### THE EVOLUTION OF PAEDIATRICS

Such speculation stems from Malthus, whose work gave Darwin the idea on which the theory of evolution was based. But even evolution is evolving, and since Darwin's time we have come to realize that natural selection is increasingly being modified by human selection. Julian Huxley has pointed out that we have reached a critical phase in the development of our planet: the evolutionary process, as now embodied in man, has for the first time become aware of itself. "Evolution", Huxley wrote, "is on the verge of becoming internalized, conscious and self-directing". As I see it, the value of discussing paediatrics, or any aspect of human affairs, in terms of evolution is that we may learn from the past and become self-directing.

Primitive civilizations practised infanticide, cannibalism and ritual sacrifice. Twins were regarded as unnatural, and done away with; so were babies born with teeth, those who sneezed directly after birth, and those born on unlucky days or in stormy weather. A sense of responsibility to infants and children has developed remarkably through the centuries. We accept now that the attitude of the tribe or nation towards its children is a barometer of progress; dare I suggest that an equally reliable barometer of progress is the attitude of the nation towards its paediatricians?

"Paediatrics as a specialized branch of medicine had no real existence before the middle of the last century. Its earlier history is only a small part of the main current of internal medicine", as Garrison wrote. It was an incidental, not the main part of the professional lives of doctors. As a specialty in its own right it probably evolved because infant feeding seems a subject apart, and infants themselves are so different from adults. After the sweat and blood and terror of delivering the mother, the obstetrician could hardly be expected to continue the same intense level of interest in the product or her labour and his. To the physician who looks after adults the infant, though doubtless a mammal, has sometimes seemed subhuman. The new specialty has inevitably grown and evolved still further.

Modern paediatrics focussed first on food. But, looking back now, nutrition seems to have been unnecessarily complicated and fussy; after all, most babies thrive on most milks, and babies have an internal timing and measuring device that tells them when and how much to feed—if only we do not put a spoke in the wheel. This sort of fussiness was probably necessary in the early exploratory efforts of the eo-paediatricians, and it applied not only to food but also, for example, to the stools. It is not so long since star-gazing as a pastime was rivalled in popularity by stool-gazing.

In the early decades of this century the physical aspects of illness absorbed nearly all the energies of paediatricians. At the time this was very proper, as it still is in the hungry countries of the world. But in this country the threat of acute physical disease is now receding. Only half a century ago, when some of us were born, six or seven infants and children died for each one that dies now. In England and Wales last year fewer than one hundred children died of tuberculosis. We are now approaching the hub of physical disorder in childhood. As a result, a very interesting new situation is developing.

To our predecessors it would seem a revolutionary concept that nearly all children are now expected to live. But it goes even further. Doctors, and especially paediatricians, have now to adapt themselves to the idea that patients are expected not only to live, but to enjoy living. This is coming about on the background of a steadily rising standard of living, a materialistic background; but there is a tendency to forget that man does not live by bread alone. Going with a broader, not exclusively materialistic approach, is the implication of a mass attack on the non-physical aspects of healthy living—what we may describe in terms of psychology or social psychiatry, but from which morality and religion cannot be separated.

Can the problem be considered exclusively in medical terms, and will the answer be provided by drugs? I take leave to doubt it. What would happen to children given tranquillizers from birth onwards, to guard them against the emotional disorders of adult life? Though preventive psychiatry must offer almost inconceivable prospects for the human race, it is difficult to believe that in this field there will be equivalents to the insecticides or the antibiotics.

### THE PAEDIATRIC SUB-SPECIES IN THIS COUNTRY

The consultant paediatric services in this country are based on hospitals. We hear rumours that paediatricians are "working themselves out of a job". We are told of children's wards that have more doctors than patients, take in lodgers to keep the cots and nurses occupied, or are being used for adult patients. The situation is worth thinking about. Let us examine one contributory factor alone, the National Health Service.

In this country before the N.H.S. era consultant paediatricians were a very small sub-species, their habitat only a few of the largest towns. Since 1948 their number has increased many times over, and they have now appeared in almost every part of these islands. In consequence, nearly every child who needs it can benefit from expert paediatric advice in or near his own home. Another consequence of the N.H.S. was decentralization. In some very large cities there may be empty paediatric wards—but is this so elsewhere? I affirm that very few paediatricians have time to twiddle their thumbs or to count napkins. Certainly they have too little time for the clinical research which is so much needed, or even to sit down and think.

We need many more facts, but here are a few. More children came to my out-patient clinics last year than ever before. Admissions to the paediatric beds in the hospitals where I attend are higher than they have ever been—though it must be added that a contributory factor is that children are not kept in hospital as long as they used to be. From the whole of the South West Region, with two-thirds of a million children under 15 years of age, I have figures that show a steady increase both of paediatric in-patients and out-patients until 1955, since when the level has remained steady. This region is probably representative of many others in that there are, in my view, too few paediatricians to deal with the many disorders which are now coming into their ambit. It seems clear that the paediatric services are held at their present level only because consultants are working at, and indeed beyond, their limit; if even a few more could be appointed the number of children efficiently dealt with would increase considerably.

A detailed investigation in Newcastle, published this year by Taylor and Davidson, tells a similar story. Between 1950 and 1957 there was little change in the admissions to children's hospitals in the Newcastle area. One point that will interest Management Committees is the demonstration from the Newcastle survey that if children are nursed in units *not* under paediatric supervision the length of their stay in hospital is appreciably increased.

It is true that doctors nowadays are rightly concerned to avoid admitting children to hospital, but in some respects this commendable reluctance may be pressed too far. If they do have to come into hospital we must ask, as Spence did, "What kind of hospital?". We should think not only of technical expertise but of many less definable factors. Neither keeping the child at home, nor ease of visiting if the child is admitted to hospital, can compensate for unnecessarily long illness, for avoidable physical or emotional sequelae, or for a dead child.

### ENVIRONMENTAL FACTORS IN PAEDIATRIC EVOLUTION

*Paediatrics abroad*

Ireland has no snakes. Australia has kangaroos. Evolution on an island may proceed in a direction very different from that on large land masses, and we live on an island. In this country, for example, general practice has always been important, and we continue to believe in the patient-doctor relationship. But in some countries of Europe general practitioners are becoming as extinct as the dodo; they are being ousted by specialists, or they are succumbing to the modern medical disease—polyclinicosis. This evolutionary trend is not one that we should encourage here.

If we look further afield we see that in the U.S.A., for example, the evolutionary process differs from our own. Is it because of the extremely rapid growth of the population? In the U.S.A. there were 40 million children in 1940, and 57 million in 1957. Or is it because of the difficulties in maintaining a permanent patient-doctor relationship with so mobile a population? Or because of the widespread conviction that the new must be an improvement on the old? We have learned in other fields of knowledge not to ignore the Russians, and in paediatrics they must certainly be taken into account. Here I must draw on what I have read, for while my views on many countries in Europe and on North America are to some extent first-hand those on Russia are culled from others, a defect which I long to remedy. Perhaps the most impressive aspect of paediatrics in Russia is that emotional difficulties, psychosomatic disorders and neuroses seem so rare. Russian doctors use a Pavlovian rather than a Freudian approach to explore the workings of the mind, and education seems to be based on conditioning and the moulding effects of the community. But please observe that the child in Russia is the most important asset of the community, by edict of the Central Committee of the Communist Party. We need to know more about paediatrics not only in countries but in cultures other than our own.

*Paediatrics in this country*

Coming back to this island, there are many factors in our changing environment to which paediatricians must adapt. An obvious one is the increasing instruction of the public in medical matters by the press and the ubiquitous television. More fundamental factors are the uncertainty that is at present confusing educationalists; the watering-down of religious beliefs; the growing view that leisure, for women as for men, is a right and not a privilege; and the increasingly high standard of material living and of mechanization. All these indirectly affect medical practice. In medicine we have also the changing pattern of nursing and nurses; the trend away from the hospital to the home; and of course the National Health Service, with its many good features but its emphasis on disease rather than health, and its deplorable schism between general practice and hospital practice.

*The Welfare State*

Many of the factors that form the environment of paediatrics can be grouped under the heading of The Welfare State, with all that this implies both for good and for bad. In the Newsholme Lectures (1953) Moncrieff described how "The Welfare State has emerged by an evolutionary process in the sphere of social affairs but without sufficient education (or propaganda) to take the people along with all its implications", and in consequence "parents demand as a right to have everything done, and the next stage is almost a refusal to do anything themselves".

Let me select a few points from a paper by Richter published this year under the provocative title of "Rats, Man and the Welfare State". He quotes first some calculations about mechanization which came as a revelation to me. In the U.S.A. in 1939 the work performed by machines was equivalent to a hundred slaves per head of the population. In this country it was only fifty slaves per head. We are obviously over-worked. Nevertheless we compare well with other countries, such as China with three or four slaves for each person. Like others before him Richter goes on to speculate about the incidence of incurable diseases and defective health (mental and physical) associated with civilization. To attempt an answer he compares men with rats—this has been done before—and wild with domesticated rats. With the transmission of mutations in the rodent welfare state, domesticity has permitted the survival of toothless rats, hairless rats, jaundiced, anaemic, blind, wobbly and waltzing rats.

The Welfare State should not however be blamed for developments that are attributable to civilization or to the progress of medical science. Nevertheless it is certainly true that in civilized countries, and particularly under a welfare state, many live who would otherwise die. In the domain of paediatrics we are responsible for the survival of many premature babies, rhesus babies, babies with congenital malformations, and others. Moreover, they may go on to reproduce their own defects in their offspring. Yet we must set against this the vast multitude of children who would have died, or would have limped through life, but have instead been given the opportunity of leading a full and healthy existence.

In the campaign against nature mankind is sheltering more and more behind the shield of medicine. As doctors we may feel flattered; as students and manipulators of evolution we may feel scared—but we should also be stimulated.

### INTERNAL FACTORS

Since doctors are increasingly playing a part in modifying the evolutionary process we should study not only our environment but also ourselves.

In biological evolution migration is important; to some extent the environment is not merely accepted but selected. In paediatrics, similarly, some of the most active, as well as the most frustrated, among us tend to explore and to move on to promising new territories. They tend also to cross-breed, and in paediatrics I should like to encourage wholeheartedly the "hybrid vigour" that results from selective cross-breeding. In various sub-species of medicine there has been a tendency to in-breeding, with breeding centres at some well known institutes claiming to produce the only true thoroughbreds. Fortunately this tendency is becoming a thing of the past, for the thoroughbred is ill-suited to adapt to changing circumstances.

One point on which the moderns differ from Darwin is that his emphasis in evolution was entirely on competition; he neglected the opposing principle of co-operation in a species or sub-species, which we now know to be important. On the whole paediatricians co-operate rather than compete, especially since the inception of the National Health Service, with advantage to themselves, to the medical profession as a whole, and to their patients.

So much for some of the evolutionary factors that we can see at work; but many others that will affect ourselves or our patients are unpredictable. Thus in our Western civilization the onset of puberty is occurring earlier and earlier in the lives of children in successive generations; if this trend continues it may change not only medicine but civilization itself as we now know it.

### THE FURTHER EVOLUTION OF PAEDIATRICS

In his *Descent of Man* Darwin discussed biological evolution in the past; Galton followed him by venturing into the future. I lay no claim to being a paediatric Galton, but it is stimulating to try to forecast, even though "we do but learn today what our better advanced judgements will unteach tomorrow".

In the near future attention in paediatrics is likely to be focussed more sharply on critical periods in the child's development. Puberty, for example, is now receiving long delayed attention; but, in the main, the focus is progressively shifting back in time. We are learning to read the child's palimpsest. We are exploring back beyond infancy, beyond the critical first breath of life, to intra-uterine existence; and even further back, to microscopic chromosomes and mascroscopic family units.

The current trend to prevent disorder rather than to "cure" will be intensified. Accidents to children in the home and on the street are already being studied from this viewpoint. More and more disorders of infancy and of later life will be prevented as obstetricians become, in Professor Neale's stimulating term, "ante-natal paediatricians"; and as we pay increasing attention to parental and family wellbeing in all its dimensions. Now that so much progress has been made in the prevention of physical disorders, Public Health Departments are commendably turning their attention to mental health, a welcome transition which has been crudely summed up in the phrase "from drains to brains". The trend towards prevention of non-physical ailments is only now discernible, but will surely be strengthened.

What of specialization? We may remind ourselves that in biological evolution specialization is a necessity, though over-specialization may lead to extinction. Will specialists suffer the fate of the sabre-toothed tigers? You will recall that sabre-toothed tigers were believed to have died out through over-specialization; though, alas, it now seems that their extinction was not due to their teeth. The moral, however, is clear, and it is as well that paediatricians in general have evolved as consultants rather than specialists. They are unlikely to become extinct so long as they remain adaptable. No one person can any longer be expert in every aspect of paediatrics; but there will surely be a continuing need for paediatric consultants with a basic grounding in all aspects of growth and with an ability to mainain perspective.

I believe, however, that neither adaptability nor perspective can be achieved by living in ivory towers—though this is hardly an apt term for the primitive buildings in which many of us work. Paediatricians must surely go into the homes and the schools; they must study children in their natural environment. And, as some are already doing, they must try to break down the barriers between hospital doctors and family doctors and public health doctors—barriers of opportunity and of practice. We are all concerned with children. We are all, directly or indirectly, helping to mould what our American colleagues call the New Paediatrics, which embraces every aspect of childhood and is not restricted to "diseases".

What of the more distant future? There will, of course, be new diseases. I suspect that many of them will lie in the province of philosophy, where doctors have always found a congenial niche. Let me foreshadow only the substitution of deficiency diseases by "excess diseases". Having seen children of school-leaving age already needing a complete set of artificial teeth, and learning from my colleagues' pointed remarks that obesity in adults shortens life, I mention concentrated carbohydrates as one example of the excess diseases against which man may need to be protected.

I fancy that one can foresee a Brave New World in which the Malthusian problem will no longer exist. There may come a time when none but the best potential parents will experience the desire to bear children. In predetermined numbers and sex, children will be born painlessly and atraumatically; and, following recent veterinary experiments, not singly but in litters. Few will suffer from congenital malformations, for these, like cancer, will have been virtually eliminated by the application of genetics and biochemistry. They will come into a world where there will be less need for surgeons—these carnivores will have to adapt themselves to a predominantly vegetarian diet. Nor will the genetically and biologically minded psychiatrists have so much to do, especially if stress, as I dare to hope, is controlled and used as the mental equivalent of muscle building. Our remote heirs will enjoy a long life before degenerative processes can no longer be profitably postponed. I foresee a time when there will be little work and much leisure for the doctor-philosophers who deal with adults—once paediatricians have learned to do their job properly.

In the centenary year of Darwin's *The Origin of Species* it has been stimulating for me, and I hope not uninteresting for you, to consider in evolutionary terms the development of paediatrics, as one part of the profession to which we are all debtors. I do not conclude by suggesting that the Ministry of Health be replaced by a Ministry of Evolution—at least, not yet. But if we ponder on evolution in the past and the present we are the more likely to adapt successfully in a fast-changing world, and to direct our future rationally and purposefully.

My views have been deliberately optimistic, as befits a doctor of children who is inevitably and pleasantly influenced by his patients. You were not meant to agree with all my forecasts; but you will agree that while children continue to be born paediatrics will continue to be practised, and that paediatrics will always be worth-while—for, "As the twig is bent so the tree will grow".

